# A qualitative approach to applying virtual reality relaxation for patients with psychiatric problems: Focus group study with healthcare professionals

**DOI:** 10.1371/journal.pdig.0001525

**Published:** 2026-07-30

**Authors:** Lisanne M. Robbemond, J. W. H. Mathijs Nijland, Zinzi E. Pardoel, Catheleine M. G. van Driel, Wim Veling

**Affiliations:** 1 Department of Psychiatry, University of Groningen, University Medical Center Groningen, Groningen, The Netherlands; 2 Department of Health Sciences, University of Groningen, University Medical Center Groningen, Groningen, The Netherlands; 3 VRelax B.V., Groningen, The Netherlands; Army Medical University, CHINA

## Abstract

Virtual Reality (VR) is increasingly being used in mental healthcare settings. VR relaxation appears feasible, effective, and acceptable for reducing stress in patients with psychiatric problems. Understanding healthcare professionals’ perspectives is essential for sustainable integration, as they play a key role in its adoption and use. This study explores the facilitators and barriers perceived by healthcare professionals in using VR relaxation (VRelax) while treating patients with psychiatric problems to support its implementation in mental healthcare, as part of a preliminary qualitative investigation informing the design of a subsequent randomized controlled trial (RCT) on its implementation and cost-effectiveness. From November to December 2021, three focus groups and three individual interviews were conducted with healthcare professionals who had used VRelax for four weeks in clinical practice. Participants were recruited via the researchers’ professional network. Semi-structured interviews were analyzed thematically. The Consolidated Framework for Implementation Research (CFIR) guided data structuring and interpretation. Fifteen nurse specialists, psychologists, and psychiatrists were included. Six themes and seventeen subthemes emerged. Key barriers included the need for evidence-based information and technical and logistical challenges. Professionals’ attitudes were shaped by their interest in technology and perceived technical skills, affecting their confidence in using VR. Healthcare professionals base VRelax use on patient characteristics rather than DSM-5 diagnoses. No themes emerged in the outer setting or implementation domains of the CFIR, suggesting underexplored systemic factors. Future implementation efforts should involve policymakers and implementation experts for a broader, multi-stakeholder approach to address these gaps. Structured guidance and clear workflows are recommended to support scalable and context-sensitive integration of VR relaxation in mental healthcare.

## Author summary

In recent years, virtual reality (VR) has emerged as a promising tool in mental healthcare. In our study, we explored how healthcare professionals experience using a VR relaxation tool called VRelax with patients who have psychiatric problems. We wanted to understand what helps or hinders its use in clinical practice, as this insight is crucial for successful and sustainable implementation. To do this, we spoke with nurses, psychologists, and psychiatrists who had used VRelax for four weeks. Their feedback revealed both enthusiasm and concerns. While many saw potential benefits for patients, they also highlighted the need for clear evidence, better technical support, and structured workflows. Interestingly, professionals chose to use VRelax based on individual patient needs rather than formal diagnoses. Our findings suggest that broader involvement, from policymakers to implementation experts, is needed to make VR relaxation a standard part of mental healthcare. This work lays the foundation for a larger study on how effective and cost-efficient VRelax can be in real-world settings.

## Introduction

Virtual Reality (VR) is increasingly used in mental healthcare settings. VR is an innovative and immersive method comprising computer-generated simulations experienced via a head-mounted display. A commonly used form is VR relaxation, which focuses on stress reduction by providing virtual natural environments. The immersive nature of VR makes it an application that promotes stress reduction without much effort from the user [1,2]. Compared to control or comparison conditions, VR relaxation is effective, feasible, and acceptable in both the general population [3] and individuals with mental health conditions [4]. However, challenges such as technological accessibility, cost-effectiveness, and varying levels of user acceptance impact the implementation of VR applications [5,6]. Without optimal implementation of VR relaxation applications, these applications may not be used as intended and may not lead to the expected improvement in patient outcomes. Exploring healthcare professionals’ perspectives is critical for the sustainable integration of VR relaxation, as these play a pivotal role in its daily use and acceptability in clinical practice [7]. This study explores the barriers and facilitators in implementing a VR relaxation application, VRelax, as experienced by mental healthcare professionals.

Despite the promising potential of VR relaxation, research on its implementation within mental health settings remains limited. Previous studies described user experiences of healthcare professionals with a focus on VR exposure therapy [8–10] rather than VR relaxation. Additionally, existing studies mostly gathered data from participants with limited experience in using VR [11] or relied on questionnaires instead of qualitative research methods that explored in-depth experiences [12]. In general healthcare, several barriers were identified, such as a lack of time and expertise experienced by the healthcare professional while using VR, insufficient knowledge of the added value and scientific evidence of VR applications in treatment, and the desire for more personalized VR applications tailored to patients’ needs and treatment goals [13,14]. Also, frequently discussed challenges include the unavailability of practical resources and a perceived lack of experience in working with VR [5]. Other implementation studies highlight that barriers such as training needs, insufficient clinical protocols, and resistance to technological innovations remain critical challenges for integrating VR into routine care [6,15].

Different implementation frameworks can be used to gain more insight into implementation processes. A commonly used framework is the Consolidated Framework for Implementation Research (CFIR) which allows for an in-depth analysis of key factors at multiple levels of implementation [15,16]. The updated CFIR consists of five domains. The *innovation domain* describes the “tool” or intervention being implemented, independent of the implementation strategy. The *inner setting domain* is the setting in which the innovation is implemented, e.g., a hospital. The *outer setting domain* explores the broader setting including the healthcare system, society, or country. The *individuals domain* describes the roles and characteristics of individual stakeholders, and finally, the *implementation process domain* describes the activities and strategies used to implement the innovation [16]. The CFIR has been applied to investigate the implementation of different digital interventions such as telemedicine [17–19] and internet-delivered CBT [20]. To date, one study applied the CFIR framework to the implementation of VR applications in mental healthcare, concluding that an iterative, agile, and systematic approach is required for the development of coherent, multi-level implementation interventions, tailored to specific contexts [7]

The current study aims to provide insight into an initial evaluation cycle of VR relaxation in mental healthcare by investigating the perspectives of mental healthcare professionals. We conducted focus groups and individual interviews with professionals who got to use a VR relaxation application, called VRelax, in clinical practice. VRelax promotes relaxation and anxiety reduction by providing immersive 360-degree videos of nature experiences with embedded slow gaming elements and relaxation exercises. Users can explore different landscapes with binaural nature sounds. The effectiveness of VRelax has been studied several times, showing that VRelax is effective in decreasing stress levels in different populations [3,4,21–23]. The studies found that most users found VRelax easy to use [21,22] and only reported a few (technical) challenges [22]. The main focus of this study was to identify facilitators and barriers experienced by mental healthcare professionals in the use of VRelax while treating psychiatric patients. The CFIR framework was used to structure the data and gain deeper insights into implementation processes at multiple levels.

## Methods

### Study setting and design

This study represents the first phase in a series of investigations of VR relaxation (VRelax) in mental healthcare. It comprises of two qualitative studies, one focusing on patient experiences [22], and the other one on mental healthcare professionals (reported here). Both were used to inform the design of a subsequent randomized controlled trial (RCT) evaluating the implementation and cost-effectiveness of VRelax across diverse mental healthcare settings.

The current study involves the experimental use of VRelax by mental healthcare professionals in clinical practice, after which semi-structured focus groups and individual interviews are conducted to identify barriers and facilitators of implementing VRelax in mental healthcare. Focus groups are used to explore specific issues, whereby the group interactions are key to the data [24]. This study design was chosen because it fosters interactions between the participants, leading to in-depth insights into what people think, and why they think what they think. As the data collection occurred during the COVID-19 pandemic, individual interviews were conducted with participants who experienced symptoms resembling COVID during the planned focus groups. The same interview guide was used for the focus groups and the individual interviews.

The Medical Ethics Review Board (METc) of the University Medical Center Groningen approved this study as exempt from the Medical Research Involving Human Subjects Act (WMO) (METc no: 2021/297). This study was conducted following the Declaration of Helsinki. Verbal and written informed consent was obtained from all participants. Study reporting was based on the COREQ-guidelines [25].

### Participants

All participants were healthcare professionals who worked in mental healthcare as psychiatrists, psychologists, or nurse specialists and worked with patients with burnout complaints, depression, anxiety, psychosis, or bipolar disorder. Participants were recruited through the network of researchers and healthcare professionals involved in this study from one academic hospital and two general mental healthcare organizations. Initially, researchers contacted colleagues within their professional network at these organizations. These contacts then forwarded the recruitment email to mental healthcare professionals within their respective institutions. Professionals who indicated to have interest in participating in the study were contacted by the researchers and were included after obtaining informed consent.

### Materials

#### VRelax.

**Fig 1:**
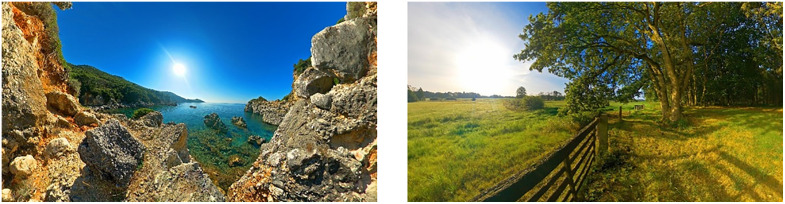
Impression of virtual natural environments in the VRelax app (source: VRelax.com).

VRelax (version 1.1.0) is a VR relaxation application created by VRelax BV. VRelax, on the Oculus Quest 2 (Meta, California, USA), provides users with an immersive 360–degree audio-visual nature experience, including more than 40 videos, such as walking in a forest or watching a sunset on a beach (see Fig 1). VRelax includes interactive gaming elements, such as popping animated air bubbles and relaxation exercises embedded in natural environments, including guided meditation and breathing exercises. Navigation within the VRelax application is done with head movements that can activate hotspots visible in virtual natural environments.

### Study procedures

The focus groups and individual interviews were conducted from November to December 2021. Potential participants received information about the study by e-mail. More detailed information was given to all professionals when they indicated that they were interested in participating. After inclusion in the study, a physical meeting was planned with one of the researchers to complete a demographic questionnaire and receive instructions on Oculus Quest 2 (Meta, California USA) with VRelax installed. Participants received instructions on how to use the headset and how to navigate through VRelax, and got a quick overview of the content. Participants were asked to use VRelax for a period of four weeks in the treatment of patients with stress complaints and a diagnosis of depression, anxiety, psychosis, or bipolar disorder. They were instructed to use VRelax in their daily practice with at least two patients. During that period, they also had the opportunity to use VRelax themselves. The goal of the study, to explore factors influencing the implementation of VRelax in clinical practice, was explained to all participants. After these four weeks, participants returned the Oculus Quest 2 to the researchers, after which the focus groups or interviews were conducted. Two focus groups (FG1 and FG2) took place in the academic hospital. The third focus group (FG3) was held at a mental healthcare organization. The individual interviews were conducted either in person or via telephone. The focus groups lasted 60–120 minutes, and interviews lasted 15–30 minutes. During the focus groups and interviews, several summaries were given by the moderator to validate the responses of the participants. All focus groups and interviews were audio-recorded and transcribed verbatim.

#### Interview guide and data collection.

A semi-structured interview guide with open-ended questions was conducted (see S1 Appendix) and used to facilitate the discussion. The interview guide was tested in a pilot session and was designed to give a broad insight into the experiences of the healthcare professionals. The interview questions were based on existing literature of VR implementation in healthcare, centering on the clinical indicating process, work processes, positive and negative experiences while using VRelax, and potential improvement of VRelax and implementation factors.

The focus groups were moderated by a female psychologist (first author) and a male psychiatrist (second author), both also working as researchers. A minimal prior relationship was established between the moderators and participants before the focus groups. The moderators were experienced in leading focus groups. The second author did the individual interviews. Only the participants and moderate were present for the focus groups or interviews. Before and during data collection, a consensus session was held between the first author, second author, and fourth author to maintain a consistent interview format. Following the third focus group and third interview, another consensus meeting was held to evaluate the findings and data saturation. The absence of new themes in the last focus group and interview led the research team to conclude that data saturation had been achieved.

### Data analysis

For the data analyses, all audio recordings were transcribed using Amberscript (*Amberscript*, 2024). Atlas.ti version 24 (*ATLAS.Ti Scientific Software Development GmbH*, 2024) was used for coding all transcribed data. First, all data was anonymized before being coded by a second author. During the coding process, a consensus meeting took place between the first author, the second author, and the fourth author. Any discrepancies were discussed until an agreement was reached. Thematic analysis as described by Braun and Clarke [26] was used, consisting of six steps: becoming familiar with the data, generating codes, generating themes, reviewing the themes and lastly defining and naming the themes and producing the report. The themes were identified by the first and second authors and discussed with third and fourth authors. Quotes were translated from Dutch to English by second author and reviewed and checked by first, third, and fifth author. The five CFIR domains were used to give a structured overview of the themes.

## Results

### Sample

Fifteen healthcare professionals participated in three focus groups and three individual interviews together. (see Table 1). Most were female (80%) and worked as a psychologist (60%).

**Table 1:** Characteristics of the focus group (FG) and individual interview (I) participants.

Characteristics	FG1 (n = 4)	FG2 (n = 3)	FG3 (n = 5)	I1	I2	I3
Female sex, n (%)	4 (100)	2 (66)	5	1 (100)	1 (100)	1 (100)
Profession, n (%)						
Psychiatrist	-^a^	1 (33)	2 (40)	–	1 (100)	–
Psychologist	4 (100)	2 (66)	1 (20)	1 (100)	–	1 (100)
Nurse specialist	–	–	2 (40)	–	–	–

^a^Empty cells indicate values that are not applicable.

### Thematic analyses

In total six themes were described, with a total of twenty subthemes. One theme was identified as a facilitator: the broad applicability of VRelax. Two barriers were identified: more evidence-based information on clinical application is needed and logistical considerations. Three themes emerged as both facilitators and barriers: technical considerations, patient characteristics influencing the indication for use, and healthcare professionals’ attitudes.

No themes emerged within the *outer setting domain* of the *implementation setting domain*. The healthcare professionals interviewed focused primarily on individual and organizational-level factors rather than external policy or structured implementation strategies. No participants explicitly mentioned external institutional barriers such as funding constraints or organizational buy-in as major factors influencing VRelax implementation. For an overview and description of the themes and subthemes, see Table 2. Illustrative quotes of the participants are provided for each subtheme. After each quote, the following characteristics are presented successively: quote number (Q), participant number (P), gender, and profession.

**Table 2:** Description of themes.

Theme	Construct name	Description	Subtheme
**I. Innovation domain**			
1. Broad applicability VRelax (F)	-Innovation Adaptability - Innovation Relative Advantage	VRelax can be used across various psychiatric problems and as a replacement for current relaxation methods or an addition to the treatment	a. Transdiagnostic approach (F) b. Alternative to treatment (F)
2. Lack of evidence-based information (B)	-Innovation Evidence-Base	The lack of evidence-based information such as expert opinions and data from recent research on how to use VRelax in an evidence-based way	a. Timing of intervention unclear (B) b. Unknown who will benefit (B) c. No information on aftercare (B)
3. Technical considerations (B/F)	- Innovation Design - Innovation Complexity	The perceptions that healthcare professionals have regarding the hardware, software, and supportive materials.	a. Clear manual and technical support (F) b. Usability of software and hardware (B/F)
**III. Inner setting domain**			
4. Logistical considerations (B)	- Structural Characteristics - Relational Connections	Healthcare professional experienced different logistical problems in their work setting which hindered the implementation of VRelax.	a. Lack of integrated distribution system (B) b. Extra time needed for explanation of VRelax (B) c. Unclarity role and responsibility (B)
**IV. Individuals domain**			
5. Patient characteristics influence indication for use (B/F)	- Innovation Deliverers - Innovation Recipients	The healthcare professional made assumptions on who is eligible for the use of VRelax and who is not, based on different patient characteristics.	a. Affinity with technology (B/F) b. Affinity with mindfulness (B/F) c. Age (B/F) d. Stress and emotion-regulation (B/F) e. Patient acceptance (B/F)
6. Healthcare professionals’ attitude (B/F)	- Innovation Deliverers	Healthcare professionals’ personal experiences, curiosity, confidence or insecurity, and concerns in the use of VRelax	a. Personal interest (F) b. assumed technical skills (B/F)

B: barrier.

F: facilitator.

### I. Innovation domain

#### Theme 1. Broad applicability of VRelax.

a Transdiagnostic approach (F)

Across all participants, there was a consensus that VRelax has a transdiagnostic applicability. One participant noted that almost all psychiatric patients experience tension, which indicates that nearly every patient might benefit from using VRelax. Participants agreed that indication is based on the level of experienced tension or stress, rather than on psychiatric diagnosis. They did not describe contra-indications for its use based on DSM-5 classification, even when explicitly asked. The broad transdiagnostic applicability of VRelax serves as a significant facilitator for its use.

*“Yes. I don’t necessarily mean patient groups categorized by DSM classification, (…), it’s really, very much transdiagnostic, so indeed people with anxiety, hyperarousal or overstimulation”.* [Q1, P14, male, psychiatrist]

*“Yes, I think a large part of the people we see already carry a lot of tension with them. So, I think it* [VRelax] *could actually be initiated for a lot of people”.* [Q2, P1, female, psychologist]

b Alternative to treatment (F)

Most participants mentioned that VRelax could be used as a replacement for medication or other therapeutic interventions and as an addition to existing treatments. Stress reduction can be achieved in many ways, such as cognitive behavioral therapy, meditation, yoga, or medication. Some participants stated that it can be used when a certain medication is not preferable at that moment or when a certain type of psychotherapy cannot be applied. This makes VRelax a valuable replacement in such cases.

*“I really used it with people, as a replacement for oxazepam”.* [Q3, P9, female, psychiatrist].

Participants stated that VRelax can complement existing therapies, such as psychotherapy or medication. Rather than replacing an existing treatment, VRelax can enhance therapeutic interventions, offering additional value to the existing treatment.

*“With the second client, tension regulation is really a part of the treatment; we’ve already worked on it in various ways. So I said, ‘Well, we now have, as it happens, a new method.’ So she was open to working with it”.* [Q4, P4, female, psychologist].

#### Theme 2. Lack of evidence-based information.

a Timing of intervention not clear (B)

About half of the participants, primarily psychologists, identified the need to understand the optimal timing for introducing VRelax in the treatment. The main question that arose was whether VRelax should be implemented at the beginning of treatment, or if it would be more effective at a later stage. They wanted to know from previously acquired evidence-based knowledge what the most appropriate timeline could be.

*“But that’s also where I find it interesting—at what point do you use it, then? I’d like to understand that a bit better, because otherwise, it becomes a kind of toy or something, and then you can choose VRelax”.* [Q5, P9, female, psychiatrist].

b Unknown who will benefit (B)

The participants expressed a desire to identify which patients are most likely to benefit from using VRelax. The participants sought a specific description or overview of patient characteristics that would be most suitable for VRelax. They emphasized the need for more evidence-based information to support their decision-making process regarding indication. A lack of such knowledge could potentially hinder implementation, as patients who might benefit from VRelax could be overlooked due to healthcare professionals’ unawareness of its potential advantages. Conversely, patients who are unlikely to benefit might receive VRelax, leading to negative experiences. Healthcare professionals aimed to avoid a potential mismatch between VRelax and their patients.

*“Yes, I would, I would also like to know more about who it’s for and when to use it. And I’m still totally open to it, and I also think, for example, I once saw something about it being used for social anxiety—seems really wonderful to me”.* [Q6, P7, female, psychologist].

c No information on aftercare (B)

The participants stated a need for evidence-based information on how to conclude treatment with VRelax. They questioned what steps to take when VRelax proves helpful: should the treatment be discontinued, would a replacement for VRelax be necessary, or could patients continue using VRelax indefinitely? They noted that having information on these questions could improve the implementation process of VRelax into clinic practice.

*“What if this works for her, helping her to cope with those obsessive thoughts differently, so she doesn’t get stuck in that analysis and becomes calmer? Because right now she has nothing and she’s very desperate. So if it works, and it really does work, then I have to take it away from her after a few weeks”.* [Q7, P8, male, psychologist].

#### Theme 3. Technical considerations.

a Clear manual and technical support (F)

All participants mentioned that the instruction manual and technical support were helpful in the use of VRelax. At the start of the study, the participants had the opportunity to use VRelax independently, with a researcher present to assist them in navigating the VR set and software. Most participants indicated that this hands-on experience helped them get familiar with VRelax. Additionally, each participant received a written manual detailing how to start the device and navigate the software. During working hours, a researcher was available for troubleshooting. Participants found this support very helpful, enabling them to use VRelax correctly in their treatment and thereby facilitating the implementation process.

*“There was, of course, an instruction manual that was already clear”.* [Q8, P5, female, psychologist].

b Usability of software and device (B/F)

Most participants found VRelax easy to use, noting that they did not encounter issues with getting started or finding a suitable natural environment.

*“I find the app quite user-friendly; you can easily find your way around in it”.* [Q9, P4, female psychologist].

However, some participants encountered difficulties. They struggled to locate the desired virtual environment in the applications menu and some participants reported instances where a black screen appeared, leaving them unsure of how to proceed.

*“But for example, those stars, I wanted to find those again, but I just couldn’t locate them anymore”.* [Q10, P9, female psychiatrist].

Additionally, some participants described difficulties with software of the Oculus Quest 2 itself. These included difficulties in connecting the device to their local WiFi. Er zijn stappen die patienten moeten doorlopen die een barriere kunnen vormen tot het starten met gebruik maken van VRelax.

and problems with adjusting the elastic bands for a proper fit. These challenges irritated, negatively impacting the continuous use of VRelax.

*“Good. Well, also something about the user-friendliness or lack thereof. I found it quite complicated to get it up and running.*[Q11, P14, male, psychiatrist].

### III. Inner setting domain

#### Theme 4. Logistical considerations.

a Lack of integrated distribution system (B)

Most participants reported the absence of an integrated distribution process and highlighted the potential benefit of having a lending system. They suggested a signed document to clarify that healthcare professionals are not responsible for the VR glasses in case of breakdowns or technical issues. Participants also noted uncertainty about where to store the VR sets when not in use and who would be responsible for them. Many participants agreed on the need for a designated employee to act as a contact person for issues related to VRelax. They believed that having such a role would benefit the long-term implementation of VRelax in their respective healthcare settings.

*“It would then act like a sort of librarian, lending the device out, keeping track of where everything is, and sending a reminder when needed back”.* [Q12, P8, male, psychologist].

b Extra time needed for explanation of VRelax (B)

Another barrier to implementing VRelax is the extra time required to explain VRelax to patients before they can start using it at home. Participants realized that properly instructing patients took valuable appointment time. which could not be allocated to other parts of the treatment, such as discussing the patient’s progress of assigned homework. As a result, some participants hesitated to incorporate VRelax into their sessions, fearing that the time commitment would interfere with other aspects of treatment.

*“Sometimes I’ve just decided: I’ll do this, then that, and then that headset* [VRelax] *would take up a lot of time, which could fit in, but we’re not going to do now. Because it’s not just five minutes and then we move on with what we were going to do”. – talking about when to use the VR glasses and the amount of time it cost to introduce”.* [Q13, P4, female, psychologist]

c Unclarity role and responsibility (B)

Some participants mentioned that they were not the primary treatment coordinator or lead therapist. In other words, they were not responsible for the treatment plan of a patient but did carry out the treatment. Prior to starting VRelax, it is essential to inform the treatment coordinator and obtain consent. This additional step can create delays and add complexity to the workflow, potentially discouraging healthcare professionals from incorporating VRelax into their treatment plans. The need for coordination and communication can be time-consuming and may interfere with the efficiency of the treatment process.

*“What I was still thinking about—I’d already given it to a client, and then I thought: hey, you’re not actually the lead therapist for this client, but it’s actually an intervention. Shouldn’t I have discussed this with the lead therapist first? So that’s maybe something too. Like, if we do this more often, we should include it in a step-by-step plan”.* [Q14, P4, female, psychologist].

### IV. Individuals domain

#### Theme 5. Patient characteristics influence indication for use.

During the indication process, most participants made assumptions about which patient would benefit from VRelax, based on specific patient characteristics rather than criteria described in the DSM-5. These assumptions were influenced by healthcare professionals’ previous experiences with eHealth technologies and their general interactions with patients. When patient characteristics align with these assumptions, it facilitates the use of VRelax; however, when they do not, it becomes a barrier to implementation.

a Affinity with technology (B/F)

The majority of the participants believed that VRelax was more suitable for patients who already had an affinity for digital technology, as they would adapt more quickly and easily to eHealth novelties such as VR.

*“I immediately thought of a patient (…) because he’s also a bit of a gadget freak—and mood complaints, but also ADHD and a lot of trouble relaxing. So I thought this is a kind of active way, I think, to relax, stimulating enough. So that’s why I immediately thought of him.”* [Q15, P3, female, psychologist].

*“But in all the busyness or something, you think, oh right, it’s easier with someone like that, that IT guy picks it up easily”.* [Q16, P6, female, psychiatrist].

b Affinity with mindfulness (B/F)

Participants assumed that patients who already practiced mindfulness in their daily lives would benefit more from VRelax compared to patients who do not. This assumption acts as a facilitator because it helps professionals identify and select patients who are more likely to benefit from using VRelax. However, it may lead to the exclusion of patients who do not practice mindfulness but could still benefit from VRelax. This selective approach could limit broader implementation and accessibility of VRelax for a diverse patient population.

*“More that I might have filled that in a bit myself, like people who seemed a bit sensitive to this [VRelax] to me, (…) but people who are a bit more inclined towards yoga, of which I know that, with a bit more self-reflection. The people who always continuously put the most trust in pills, I didn’t choose them in the past few weeks”.* [Q17, P9, female, psychiatrist]

c Age (B/F)

Most participants assumed that younger patients were generally more suitable for VRelax and could benefit more from it compared to older patients. This potentially could exclude older patients who can benefit from using VRelax and limiting the implementation of VRelax for a large group of patients.

*“Yes, a young person, because I thought I might not do this directly with someone around 60 or so. They might have a bit more feeling for it, and I think they mainly thought it was a really cool gadget”.* [Q18, P2, female, psychologist]

d Stress and emotion-regulation (B/F)

Some participants identified relaxation and emotional regulation challenges as distinct patient characteristics, separate from the patient’s current stress level. They observed that some patients had previously undergone various relaxation interventions without success. In such cases, participants believed VRelax might be more suitable due to its unique mechanism of action. They assumed that, since VRelax offers an alternative approach to relaxation compared to methods like medication and psychotherapy, it would be a good fit for these types of patients.

*“(…) My guess now is that it’s mainly suitable for people who struggle relax by themselves without something. To get into a certain mode. This is, of all the devices out there, the most compelling, almost, which is also a strength for those who find it very difficult to reach that state”.* [Q19, P11, male, psychiatrist].

e Patient acceptance (B/F)

Almost all participants observed that not all patients they considered suitable for VRelax were willing to try using it. Patient willingness and expectations of patients play a crucial role in the successful implementation of VRelax. Patients who easily got distracted or had unsuccessfully tried other relaxation interventions seemed like likely candidates for VRelax. Participants stated that if patients are unwilling to try it or have unmet expectations, it can hinder the adoption and effectiveness of VRelax in their treatment plans.

*“… he just gets distracted and quickly loses interest, so I thought, well, this [VRelax] is it. So I set my expectations for myself really high. Then after the first week, it turned out he hadn’t really gotten around to it, and I discussed that with him. In the second week, I hoped for a better result, but it was actually the same. So I found that really disappointing because I had genuinely hoped it would help him. At the same time, he did say, but that’s more the gadget freak in him, that he would really like to have such a headset”.* [Q20, P3, female psychologist].

#### Theme 6. Attitude of the healthcare professional.

a Personal interest (F)

Most participants were curious about using VRelax with their patients but also wanted to experience VRelax themselves, which facilitates its long-term use. Approximately half of the participants reported using VRelax to become more familiar with the content. This firsthand experience allowed them to integrate the acquired knowledge into their treatment strategies.

*“Well, I feel competent in this area because I’ve had the VR glasses for two weeks myself, so I’m somewhat of an expert by experience in using it, but as for implementing it in treatment, no, it’s purely based on my own experience that I can explain it to others”.* [Q21, P5, female, psychologist].

Additionally, most participants expressed curiosity about how to integrate VRelax into treatment. They perceived it as an innovative application that could potentially lead to positive results in treatment. This openness to using VRelax facilitates its adoption in therapeutic practices.

*“Also, just out of curiosity. How does something like that work, and how can you apply it in your therapy?”.* [Q22, P2, female, psychologist]*.*

b Assumed technical skills (B/F)

Some participants expressed concerns about their technical skills when using VRelax in the treatment. They hesitated to implement VRelax because they were unsure whether they could explain it properly to their patients or assist them if needed. Conversely, almost half of the participants felt confident in their ability to implement VRelax in their patients’ treatment, describing VRelax as an intuitive application.

*“I didn’t even know if, after the short trial, I could give the instructions correctly. So I was just glad that they both figured it out themselves. I had arranged that if it didn’t work, they would send a message that* *evening, and we’d have a call the next day. But then I did think, I hope I’ll be able to figure it out when I’m on the phone”.* [Q23, P1, female psychologist].

*“I found, I do feel competent in* [using VRelax] *(...)”.* [Q24, P14, male, psychiatrist].

## Discussion

This study aimed to identify barriers and facilitators experienced by mental healthcare professionals using VR relaxation in treatments for patients with psychiatric disorders. Six themes were identified and categorized within the CFIR. Three themes in the innovation domain: broad applicability VRelax, lack of evidence-based information, and technical considerations. One theme in the inner setting domain: logistical considerations. And two themes in the individuals domain: patient characteristics influence indication for use and healthcare professionals’ attitude. No themes were found for the outer setting domain and the implementation domain.

This study identified several key themes emerging from participants’ discussions, notably the ease of selecting patients to use VRelax due to its broad applicability. Nevertheless, patient characteristics significantly influence the selection process, potentially excluding individuals not aligning with the participant’s assumptions. The healthcare professionals viewed VRelax as a transdiagnostic application for reducing stress complaints, without excluding any DSM-5 diagnoses. The common misconception that older individuals are less technologically proficient than younger individuals, research indicated no significant difference in workload and usability while using VR between age groups [27]. Previous research did suggest potential contraindications, such as cognitive limitations, dissociation, or cybersickness [5], however, none were observed in this study. A tailored approach can mitigate potential contraindications that were not observed in this study [28].

The results of this study also emphasize that evidence-based information and technical and logistical considerations influence the sustainable implementation of VRelax. Similar challenges are observed when implementing VR, internet, and eHealth interventions in various settings [29–31]. Research suggests including professionals in the decision-making and strategy development stages to increase acceptance [31], potentially creating viable strategies for engagement and long-term implementation.

Notably, barriers and facilitators were only found in individuals, innovation, and inner setting domains, in line with the most recent study done on this topic [7]. Both studies found that investing time and effort in using VR in daily practice is an important implementation factor, a barrier when not addressed properly. Another barrier and facilitator was found in participants’ beliefs about their self-efficacy in using technology in clinical settings, which are influenced by their interest in integrating technology into their practice and their perceived technical skills, as supported by previous research [32]. Our findings differed in that the absence of goal-setting was not identified as a barrier, likely reflecting differences in implementation within rehabilitation compared to mental healthcare settings [15,32–34]. Offering opportunities to practice with the equipment outside of clinical time can help support skill development. This can result in higher self-efficacy and positively influence the implementation of VRelax in mental healthcare.

The absence of themes within the outer setting and implementation process domains of the CFIR framework, underscores a potentially critical gap in the data, raising questions about their role in this context. One explanation may lie in the clinical focus of the participants, who were mainly frontline healthcare professionals engaged in direct patient care. Their attention is drawn to factors within their immediate environment, such as patient characteristics, practical logistics, and individual attitudes, rather than broader systemic or policy-level influences. This tendency has been observed in previous research, where clinicians often view external factors such as reimbursement models, organizational strategy, or policy constraints as outside their sphere of influence [15,32]. Another possibility is that the healthcare organizations involved had not yet developed or communicated structured implementation strategies for integrating VRelax, including leadership engagement, formal planning processes, or policy alignment. Without institutional support mechanisms or visible implementation leadership, healthcare professionals may not be aware of, or actively engaged in, the broader contextual processes that support sustainable adoption [7,19]. Prior research has emphasized that system-level factors, including governance, infrastructure, and external partnerships, play a critical role in the success or failure of digital health interventions [6]. Frontline clinicians’ perspectives may not fully capture broader organizational and policy-related factors that shape implementations. Future investigations should include additional stakeholders like managers, implementation coordinators, policy advisors, and IT personnel to better understand contextual dynamics [11,19].

### Strengths and limitations

The design of the study, in which the participant received minimal instructions on how to apply VRelax, allowed for the identification of the main barriers and facilitators perceived by healthcare professionals, instead of potentially biasing their responses with detailed guidance or instructions.

Including professionals from different clinical settings and different professions enriched the data and improved the relevance across mental healthcare contexts. However, several limitations must be considered when interpreting the results. This study did not incorporate additional sources such as direct observations, which may limit the depth of insights. Additionally, the participants may represent a self-selecting group with a particular interest in digital innovations like VR, which could restrict the applicability of the findings to all professionals in mental healthcare. Finally, no barriers or facilitators were identified in the outer setting domain and implementation process domain, limiting the comprehensiveness of the implementation perspective and suggesting the need for further exploration of these domains in future research.

### Implications

Given the promising results of this study, emphasizing that VRelax is widely applicable in mental healthcare, it is crucial to develop tailored approaches that address individual patient characteristics. Additionally, combining these findings with insights from patients’ perspectives on using VRelax [22] highlights the importance of personalizing VRelax to enhance its long-term use. The findings from this study suggest that future research on VR interventions in mental healthcare should adopt a broader, multi-stakeholder approach. While clinicians offer essential insights into patient-level and workflow-related barriers, system-level factors, such as leadership engagement, funding structures, and institutional policies, remain underexplored. Including perspectives from managers, IT support staff, and policymakers may reveal critical facilitators and barriers currently overlooked. To better capture the complexity of implementation across settings, the theoretical domain framework (TDF) could be used. The TDF offers an implementation framework with 14 domains, classified as barriers or facilitators, to explain determinants of behavior change [35]. A recent study conducted interviews with clinicians and managers to explore how to implement VR in mental healthcare, identifying 11 domains of the TDF [36]. Based on their results, the implementation should focus on targeting key mechanisms of change (i.e., beliefs about consequences, knowledge, and resources) for which essential intervention functions will include education, training, environmental restructuring, and enablement [36]. However, their results are not specifically focused on VR relaxation.

Beyond individual enthusiasm, institutions should address logistical barriers, role clarity, and integration into clinical routines. The ERIC (Expert Recommendations for Implementing Change) tool [37] can serve as a practice guide in this context, offering evidence-based strategies such as identifying and preparing champions, providing ongoing training, developing formal implementation blueprints, and restructuring workflows to accommodate new technologies. Policymakers and organizational leaders should prioritize cross-disciplinary collaboration and invest in capacity building to ensure that VR interventions are embedded in both clinical and organizational infrastructures. This structured approach can help bridge the gap between promising pilot implementation and long-term implementation at scale.

As outlined in the RE-AIM framework [38], successful integration of health interventions depends on more than efficacy; it requires knowledge about real-world application, including adoption, implementation, and maintenance. While clinicians were enthusiastic, the need expressed by professionals for more structured guidance, including when to initiate VRelax, how to identify suitable patients, and what aftercare is needed. A guide covering clinical indications, care integration, and aftercare, co-developed with end-users and based on clinical evidence, could enhance consistent use. Additionally, an implementation plan, such as aligned with tools like ERIC [37] should outline logistical workflows and team roles, addressing barriers like task responsibilities. This would ensure a coherent, scalable, and context-sensitive implementation of VR relaxation in mental healthcare.

## Conclusion

We identified barriers and facilitators experienced by mental healthcare professionals using VRelax, highlighting the broad applicability of VRelax, the need for evidence-based information, the importance of addressing technical and logistical considerations, and the influence of patient characteristics and healthcare professionals’ attitudes. The absence of themes in the outer setting and implementation domains suggests broader systemic and policy-level factors may be underexplored. Future research on VR interventions in mental healthcare should adopt a broader, multi-stakeholder approach. Additionally, structured guidance and implementation plans, for example aligning with the ERIC tool, should outline logistical workflow and team roles to ensure coherent, scalable, and context-sensitive implementation of VR relaxation in mental healthcare.

## Supporting information

S1 Appendix Interview guide. (DOCX)
