# Supplementary material for: A qualitative approach to applying virtual reality relaxation for patients with psychiatric problems: Focus group study with healthcare professionals
Source: PLOS Digit Health. 2026 Jul 30;5(7):e0001525. doi: 10.1371/journal.pdig.0001525 (PMC13422827; doi:10.1371/journal.pdig.0001525)
Supplement: S1 Appendix — (DOCX) [file pdig.0001525.s001.docx]

**S1 Appendix**

**Interview Guide**

**Opening – 3 min** (Objective: introduction, creating group cohesion, emphasizing commonalities between participants)

1. What is your name? At which institution do you work?

**Introduction – 5 min** (Objective: triggering thoughts about VRelax, making participants comfortable with speaking)

1. What were your initial thoughts on participating in this panel about VRelax?

**Transition – 5 min** (Objective: focusing thoughts on concrete recollections of using VRelax over the past two weeks)

1. For which types of patients have you offered VRelax?

**Key questions – approx. 10 minutes/questions** (Objective: answering the research question)

1. When and for which patients did you consider VRelax to be applicable?
2. What considerations did you have before recommending VRelax to a patient?
3. What was it like to use VRelax in treatment? What aspects were easy to use? What challenges did you encounter while using VRelax?
4. How did offering VRelax fit into your workflow? (Was VRelax readily available? Did you have sufficient time?)
5. What do you need to structurally integrate VRelax into your working methods? Did you feel competent, or do you require additional training or instructional materials?
6. What feedback did patients provide about using VRelax? What questions did they have?
7. What positive aspects of VRelax have not yet been mentioned?
8. How can we enhance these positive aspects?
9. What negative aspects of VRelax have not yet been mentioned?
10. How can we improve these negative aspects?

**Ending – 5 min** (Objective: closing and summarizing)

1. We have about 10 minutes left. During this session, we have discussed many topics. In your opinion, which topic should we prioritize and take immediate action on?
